# Protective Effect of Resveratrol Against Corticosterone-induced Neurotoxicity in PC12 Cells

**DOI:** 10.1515/tnsci-2019-0038

**Published:** 2019-08-24

**Authors:** Ye Zhang, Yun He, Ning Deng, Yan Chen, Jiecong Huang, Wei Xie

**Affiliations:** 1 School of Traditional Chinese Medicine, Southern Medical University, Guangzhou, Guangdong, 510515, China; 2 Department of Traditional Chinese Medicine, The Third People’s Hospital of Yunnan Province, Kunming, Yunnan, 650011, China; 3 Department of Orthopedics, Calmett Hospital & The First Hospital of Kunming, Kunming, Yunnan, 650224, China; 4 Normal Human Anatomy and Histological Embryology Department, Yunnan University of Traditional Chinese Medicine, Kunming, Yunnan, 650500, China; 5 Department of Encephalopathy, Guangzhou Conghua Hospital of Traditional Chinese Medicine, Guangzhou, Guangdong, 510900, China; 6 Department of Traditional Chinese Medicine, Nanfang Hospital, Southern Medical University, Guangzhou, Guangdong, 510515, China

**Keywords:** resveratrol, PC12 cells, corticosterone, apoptosis, neurotoxicity

## Abstract

**Objective:**

Resveratrol(RES) is a natural polyphenol which possesses an anti-depressant effect. However, the mechanisms of its anti-depressant effect remain unclear. The aim of the study is to investigate the potential mechanisms in the neuro-protective efficiency in the corticosterone-induced pheochromacytoma 12 (PC12) cells.

**Methods:**

PC12 cells were treated with 200 μM of corticosterone in the absence or presence of different concentrations of RES for 24 h. Then, cell viability was measured by Cell Counting Kit-8 assay. Apoptosis of PC12 cells was measured by Annexin V-FITC and Propidium iodide (PI) labelling. The expression of apoptosis-related proteins including Bax, Bcl-2, caspase-3 was determined by western blotting.

**Results:**

The results showed that treatment with 200 μM of corticosterone induced cytotoxicity in PC12 cells. However, different concentrations of RES (2.5μmol/L, 5μmol/L and 10 μmol/L) significantly increased the cell viability, suppressed the apoptosis of PC12 cells, down-regulated Bax and caspase-3 protein expression, and up-regulated Bcl-2 protein expression, compared to the model group (*p*<0.05).

**Conclusion:**

Resveratrol has a protective effect on corticosterone-induced neurotoxicity in PC12 cells, which may be related to the apoptosis via inhibition of apoptosis-related proteins and displays the antidepressant-like effect.

## Introduction

Depression is one of the most common mental disorders, more than 300 million people worldwide suffering from depression. Depression leads to a decline in social ability and causes severe burden [1,2]. The pathogenesis of depression has not been fully elucidated. Some acceptable mechanisms include the monoamine neurotransmitter hypothesis, the hypothalamic-pituitary-adrenal (HPA) axis activation hypothesis, nerve-inflammation hypothesis, cytokine hypothesis [3, 4, 5]. Antidepressant drugs such as serotonin reuptake inhibitors can inhibit the activation of HPA axis [6, 7]. However, long-term use antidepressant can cause lots of side effects. The discovery of new antidepressants with high efficiency and low toxicity is one of the important research topics in psychopharmacological field. Resveratrol, known as trans-3,4,5-trihydroxy-stilbene, is a kind of biological polyphenols, mainly derived from peanuts, grapes and mulberry [8, 9, 10, 11]. Researches have been shown that RES could improve depression behavior [12, 13, 14, 15, 16]. The mechanisms may associate with the regulation of brain derived neurotrophic fatcor, HPA axis, an increase of 5-HT, reduction of inflammatory factors, neurons protection [17, 18, 19, 20].However, the exact mechanism is still unknown.

The PC12 cell line is a popular cell model which is common use in a variety of studies. It exerts typical neuron-like properties and produces glucocorticoid receptors [21]. It also has been shown that high concentrations of corticosterone can induce cellular damage of PC12 cells [22,23]. Further, the antidepressants have been demonstrated to protect against cytotoxicity induced by corticosterone in PC12 cells [24]. Research also showed that cytoprotective effect is a common action pathway for antidepressants [25].

Thurs, in the present study, PC12 cell line was first applied to investigate the neuroprotective effect of RES and its potential mechanisms.

## Materials and Methods

### PC12 cell line

Rat adrenal medullary pheochromocytoma PC12 cell line was purchased from the cell resource center of Shanghai Institutes for Biological Sciences.

### Drugs and reagents

Resveratrol (Beijing Solarbio CO., Ltd, China); Corticosterone (Dalian Meilun Biotechnology CO., Ltd, China); Polyclonal rabbit antibodies (Bax, Bcl-2, caspase-3) (Santa Cruz Biotechnology CO., Ltd, CA); Cell Counting Kit-8 **(**CCK-8) reagent (Colleague, Japan); Flow cell apoptosis assay kit (Nanjing Vazyme CO., Ltd, China).

### Cell culture and treatment

PC12 cells were maintained in DMEM/F12 medium supplemented with 10% fetal bovine serum, 200 U/mL penicillin and 100 μg/mL streptomycin. A recent study showed significant differences in reproducibility can be

conferred when variable passage numbers of PC12 cells are used [26]. The 2-5 generations of cells in good culture condition was chosen to use in our study. Cells were seeded at a density of 2×10^4^ cells/well in 96 - well culture plates or cultured in cell bottle at a density of 2×10^4^ /mL in a humidified atmosphere containing 5% CO_2_ atmosphere at 37°C for 24 h. The cells were divided into six groups: Control group, where PC12 cells were not treated; Model group, where PC12 cells were treated with 200 μM corticosterone; and RES groups with different concentrations of RES (1μM, 2.5 μM, 5 μM, 10 μM). Cells in each group were cultured for 24 h.

### Cell viability assay

Cell Counting Kit-8 **(**CCK-8) assay was used to measure cell viability. Cells in each group were seeded in 96-well plates, and followed by incubation of this cells with different concentrations of RES for 24h and then the medium was replaced with CCK-8 reagent 10ul/well at 37°C and incubated for 4h. The optical density of each well was measured using a microplate reader to determine absorbance (A). Each sample was set in 4 multiple wells and the mean value was calculated. The blank control group was the well with only the culture medium. The survival rate of PC12 cells (%) was calculated: (A in the experimental group - A in the blank control group)/ (A in the control group - A value in the blank control group) × 100%.

### Apoptosis Detection

The cells in each group were washed twice with 0.1M PBS, digested with 0.25% trypsin, and then washed by ice-cold PBS, centrifuged at 300 g at 4 °C for 5 min. According to the instruction, single cell suspension liquid was obtained and resuspended with 100μl Binding buffer and constantly shaken for 15 sec. Then, 5 μl Annexin V-FITC and 5 μl Propidium iodide (PI) was added and incubated at 4 °C for 30 mins in dark. Finally, the cells were incubated with 400 μl Binding buffer for 1 h. The flow cytometry was used to detect the apoptosis at a wavelength of 488 nm. Cell apoptosis rate was calculated.

### Western blot analysis

Western blot was used to detect the expression of apoptosis-related proteins: Bax, Bcl-2 and caspase3. Cells in each group after 0.25% trypsin digestion were centrifuged at 1000 rpm for 5 min at 4°C and washed with PBS three times. Then, Cells were lysed with ice-cold cell lysis buffer and centrifuged at 4 ℃ for 15 min. Protein concentration was measured using a BCA kit. Equal amounts (20 μg) of protein were electrophoresed on SDS acrylamide gels and then proteins were transferred to nitrocellulose membranes. 5% skim milk in TBST buffer was used to block the non-specific binding. The membranes were incubated with corresponding primary antibodies overnight at 4 ℃ and then incubated with IgG secondary antibodies for 1 h at room temperature. Finally, the detection was performed using enhanced chemiluminescence. The bands were quantified using Quantity One imaging software. The ratio between the target band and the reference β-actin was used as a semi-quantitative result.

### Statistical analysis

SPSS 20.0 statistical analysis software package was used to process the data. Data were expressed as means ± SD, and multiple group comparisons were performed using t-test. *p* < 0.05 was considered to indicate a statistically significant difference.

## Results

### RES protects against the cytotoxicity of corticosterone-induced PC12 cells

**Fig. 1:**
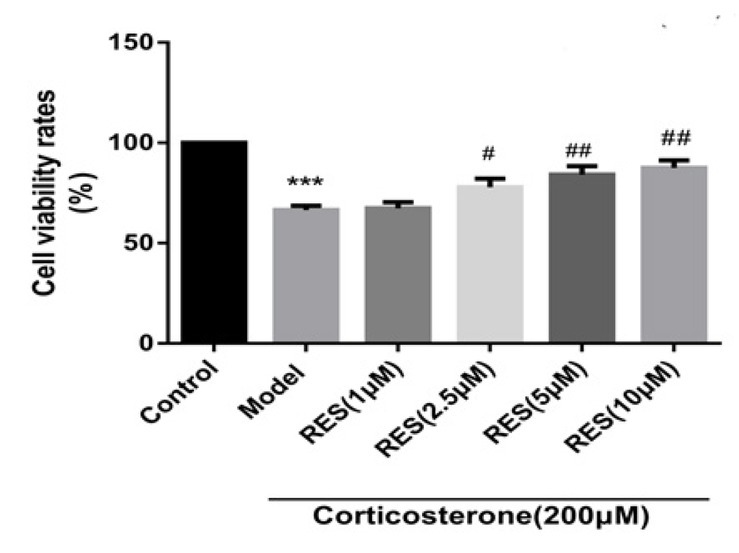
Effects of RES on cell viability rates in corticosterone-induced PC12 cells. The control group is treated without corticosterone and the model group was treated with 200 μM corticosterone on PC12 cells. Data are presented as means ± SD (n=6). ***p < 0.01 vs control group. RES groups are of different concentrations of RES (1μM, 2.5 μM, 5 μM, 10 μM) on corticosterone-induced PC12 cells. Data are presented as a percentage of model and the results were expressed as the means±SD (n=6). #*p* <0.05 and ## *p*<0.01 vs model group

Differentiated PC12 cells were treated with 200 μM of corticosterone in the absence or presence of different concentrations of RES for 24 h. Subsequently, the cell viability was measure by a CCK assay. As shown in figure 1, the cell survival rate is 65% of the model group which significantly decreased compared to the control group (*p*<0.01). Compared to the model group, the cell survival rates of different concentrations of RES (2.5, 5 and 10 μmol/L) are 73.4±0.29 %, 78.9±0.33 % and 82.6±0.23 %, respectively, which are significantly increased (*p*<0.05). However, the cell survival rate (66.9±0.24%) of 1μmol/L of RES has no significant difference (p>0.05).

### RES attenuates cell apoptosis of corticosterone-induced PC12 cells

**Fig. 2:**
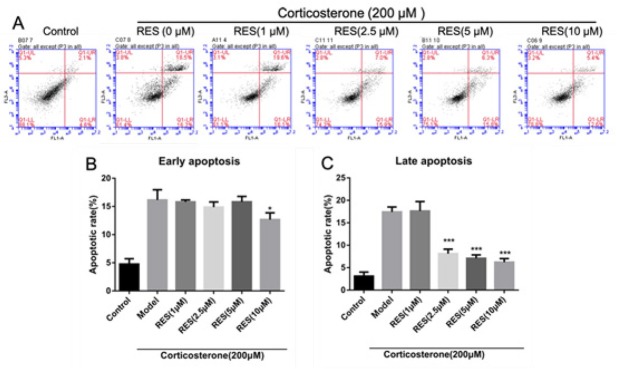
Effects of RES on apoptosis rate in corticosterone-induced PC12 cells. The effect of RES on corticosterone-induced PC12 cell apoptosis based on flow cytometry analyses. The proportion (%) of cells in each quadrant is shown. Lower left quadrant (absence of both markers) indicates viable cells; upper left quadrant [propidium iodide (PI) positive] indicates cellular necrosis; upper right quadrant (Annexin V positive and PI positive) indicates late-stage apoptosis or cellular necrosis; lower right quadrant (Annexin V positive) indicates early-stage apoptosis. (B) Analysis of early-stage apoptosis, (n = 3). (C) Analysis of late-stage apoptosis, (n = 3). Data are expressed as a percentage of the control and the results are expressed as the means ± SD. *p < 0.05 and ***p < 0.01 vs model group.

In terms of exploring the effects of RES on cell apoptosis in corticosterone-induced PC12 cells, Annexin V- FITC, PI and flow cytometry were used. As shown in figure 2 (A, B and C), the ratio of early apoptotic cells (Annexin V+/PI-) and late apoptotic cells (Annexin V+/PI+) in the model group increased to 16.7±0.52 % and 18.5±0.74%, respectively compared with the control group (*p* < 0.05). However, 2.5μmol/L RES, decreased the early and late apoptosis rate to 15.27±0.39% and 6.8±0.42 %, 5 μmol/L RES decreased to 12.7±0.33% and 5.61±0.43 %, 10 μmol/L RES dropped to 15.73±0.57 % and 6.38±0.24%, respectively (*p*<0.05).

### RES changes the expression of Bcl-2, Bax and caspase-3 of corticosterone-induced PC12 cells

**Fig. 3:**
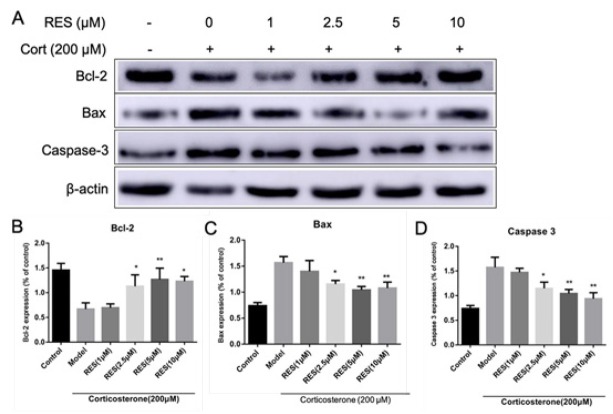
Effects of RES on the expression of Bcl-2, Bax and caspase-3 in corticosterone-induced PC12 cells. The model group was treated with 200 μM corticosterone(Cort) on PC12 cells. RES groups are of different concentrations of RES (1μM, 2.5 μM, 5 μM, 10 μM) on corticosterone-induced PC12 cells. Figure A: Western blot analysis; Figure B, C, D: Quantification of Bcl-2, Bax and caspase-3 expression were presented in bar graphs as the fold-increase, respectively.Data are as means ± SD (n=5). *p < 0.05 and **p < 0.01 vs. model group.

For further research, the effects of RES on the expression of apoptosis-related proteins were measured. As shown in figure 3, compared to the control group. the expression of Bax and caspase-3 in the model group significantly increased (*p*<0.01) while Bcl-2 expression reduced (*p*<0.01). Bax and caspase-3 expression of RES groups was down-regulated, while Bcl-2 expression increased (*p*<0.05).

## Discussion

Depression is a mood disorder characterized by persistently feelings of low self-esteem, pessimism,and despair. About 15% of patients with depression commit suicide. The pathogenesis of depression has not been fully elucidated until now. The currently accepted mechanisms include a mono amine neurotransmitter hypothesis, the HPA axis activation hypothesis, nerve-inflammation hypothesis, cytokine hypothesis, neural plasticity reduction hypothesis, the limbic system loop hypothesis, etc. The activation of HPA axis hypothesis considered that patients with depression activated pathological HPA axis excessively, which was closely associated with suicidal behavior of depression[27]. Antidepressant drugs such as serotonin reuptake inhibitors can inhibit the activation of HPA axis[28, 29].

In the study, it is found that RES increased the cell viability, reduced apoptosis, and relieved the neurotoxicity of corticosterone on PC12 cells. We investigated the expression of three apoptosis-related proteins including Bcl-2, Bax and caspase-3. Apoptosis is associated with the activation of a genetic program in which apoptosis effector genes promotes cell death. This is regulated by the action of the Bcl-2 family of proteins, which includes anti- and pro-apoptotic members such as Bcl-2 and Bax. It was reported that Bcl-2 binds to the mitochondrial membrane and undergoes competitive binding with Bax, forming the Bcl-2/Bax heterodimer, thereby, leading to inhibiting apoptosis. As a pro-apoptotic molecule, Bax can be combined into a Bax-Bax homodimer to form the composition of the mitochondrial membrane permeable channels, through which cytochrome C can transfer from mitochondria into cytoplasm which activate the caspase-related apoptosis cascade, resulting in mitochondrial-dependent apoptosis [30, 31, 32, 33].

In order to further explore the molecular mechanism of RES in inhibiting corticosterone-induced PC12 cell apoptosis, Bcl-2, Bax and caspase-3 proteins were detected by western blot in this study. The results showed that the concentrations of resveratrol range from 2.5 to10 μmol/L inhibit apoptosis, increase Bcl-2 protein expression, reduce Bax and Caspase 3 protein expression, which indicated that the regulation of RES on neuron apoptosis is mainly by increasing the ratio of Bcl-2 / Bax and inhibiting the activation of Caspase pathway.

We investigate the potential cytoprotective mechanism of RES. As a small molecule, RES is expected to pass the plasma membrane and accumulate within cells. When PC12 cells were stimulated by corticosterone, a large amount of reactive oxygen species (ROS) was produced. These excessive ROS would damage the mitochondria and cytomembrane, leading to apoptosis. Due to the antioxidant effect, RES suppressed oxidative stress, reduced the content of ROS. Moreover, we also found that RES could inhibit mitochondrial apoptotic pathways by reducing Bax and caspase-3 expression and increasing Bcl-2 expression. The results of our study showed that RES is closely related to mitochondrial pathway, but whether it is associated with other regulation pathways still needs further investigations.

In the study, we first demonstrated that RES exerts neuroprotective effect in the corticosterone-induced PC12 cells and its mechanism may be related to the apoptosis-related proteins.
